# Human rheumatoid arthritis tissue production of IL-17A drives matrix and cartilage degradation: synergy with tumour necrosis factor-α, Oncostatin M and response to biologic therapies

**DOI:** 10.1186/ar2772

**Published:** 2009-07-23

**Authors:** Ellen M Moran, Ronan Mullan, Jennifer McCormick, Mary Connolly, Owen Sullivan, Oliver FitzGerald, Barry Bresnihan, Douglas J Veale, Ursula Fearon

**Affiliations:** 1 Department of Rheumatology, St. Vincent's University Hospital, Dublin Academic Healthcare and The Conway Institute of Biomolecular and Biomedical Research, Elm Park, Dublin 4, Ireland

## Abstract

**Introduction:**

The aim of this study was to examine IL-17A in patients, following anti-TNF-α therapy and the effect of IL-17A on matrix turnover and cartilage degradation.

**Methods:**

IL-17A expression was examined by ELISA and immunohistology in the rheumatoid arthritis (RA) joints. RA whole synovial tissue explant (RA ST), primary synovial fibroblasts (RASFC), human cartilage and chondrocyte cultures were stimulated with IL-17A +/- TNF-α and Oncostatin M (OSM). Matrix metalloproteinase (MMP) and tissue inhibitor (TIMP-1) were assessed by ELISA and zymography. Cartilage proteoglycan release was assessed histologically by Safranin-O staining. Clinical parameters, IL-17A, MMP/TIMP were assessed in patients pre/post biologic therapy.

**Results:**

IL-17A levels were higher in RA vs osteoarthritis (OA)/normal joints (*P *< 0.05). IL-17A up-regulated MMP-1, -2, -9, and -13 in RA ST, RASFC, cartilage and chondrocyte cultures (*P *< 0.05). In combination with TNF-α and OSM, IL-17A shifted the MMP:TIMP-1 ratio in favor of matrix degradation (all *P *< 0.05). Cartilage proteoglycan depletion in response to IL-17A was mild; however, in combination with TNF-α or OSM showed almost complete proteoglycan depletion. Serum IL-17A was detected in 28% of patients commencing biologic therapy. IL-17A negative patients demonstrated reductions post therapy in serum MMP1/TIMP4, MMP3/TIMP1 and MMP3/TIMP4 ratios and an increase in CS846 (all *P *< 0.05). No significant changes were observed in IL-17A positive patients.

**Conclusions:**

IL-17A is produced locally in the inflamed RA joint. IL-17A promotes matrix turnover and cartilage destruction, especially in the presence of other cytokines, mimicking the joint environment. IL-17A levels are modulated *in vivo*, following anti-TNF therapy, and may reflect changes in matrix turnover.

## Introduction

Rheumatoid arthritis (RA) is a common autoimmune disease characterised by proliferation of synovial tissue (ST) and joint erosion [1]. Angiogenesis is an early, critical event enabling lymphocytes and macrophages to enter the joint cavity by active recruitment via the endothelium [2]. New vessels and leukocyte migration lead to expansion of the ST into an aggressive tumour-like pannus. The hyperplastic ST fibroblasts (RASF) of the lining layer invade the cartilage causing degradation via proteolytic cleavage of aggrecan and collagen [3]. Cytokines and growth factors are required to stimulate cell survival, proliferation and extracellular matrix (ECM) degradation as part of this process [4]. Cartilage and bone degradation is characterised by a loss of ECM through activation of matrix metalloproteinases (MMPs) and decreased production of specific tissue inhibitors such as tissue inhibitor of metalloproteinase 1 (TIMP-1) [5]. Joint destruction can be visualised radiographically and is associated with long-term functional disability [6,7]. Cartilage turnover can also be monitored by measuring synthesis and degradation products of cartilage-specific collagens and proteoglycans [8]. Recent studies by our group and others have demonstrated that these collagen biomarkers can be used to monitor disease activity and predict radiographic outcome in patients with inflammatory arthritis [9].

Targeted biologic therapies including anti-TNF-α have advanced the treatment of inflammatory arthritis. Some patients, however, do not respond, highlighting the need for new therapeutic targets. The pro-inflammatory cytokine IL-17A is one such potential target. IL-17A is the first identified member of the IL-17 family (A to F), and it is most closely related to IL17F with 50% sequence homology [10]. IL-17F demonstrates similar but less potent effects to IL-17A [11]. The recently identified subset of T helper cells termed Th17 cells are the main source of IL-17A. However, CD8+ T cells, γδ T cells and natural killer T cells can also secrete IL-17A. In both the humans and mice, differentiation of naïve T cells into Th17 cells involves the cytokines transforming growth factor (TGF)-β, IL-6, IL-21, IL-1β and IL-23 [12].

It has been shown previously, in RA ST from joint replacement surgery, that IL-17A is spontaneously produced; also high levels have been demonstrated in the synovial fluid (SF) of RA patients. IL-17A has also been detected in SF from osteoarthritis (OA) patients; however, levels were lower than in RA SF [13,14]. *In vitro*, IL-17A stimulates the production of cytokines and chemokines including TNF-α, IL-1, IL-6 and IL-8 [15-17]. IL-17A also up-regulates MMP expression by chondrocytes and synoviocytes resulting in cartilage damage [18,19] Furthermore, IL-17A causes an upregulation in RANKL production leading to bone erosion [20].

In animal studies *in vivo *a role for IL-17A has been established in mediating cartilage and joint damage [21]. Injection of IL-17A, alone, into naïve murine knee joints resulted in extensive cartilage depletion and bone erosion [22]. Continuous administration of IL-17A by gene expression in mice significantly increased inflammatory infiltrate, cartilage and joint erosion [23]. Inhibition of IL-17A using blocking antibodies and a soluble receptor in a mouse model or with IL-4 does protect against inflammation and bone damage [24]. A role for IL-17A in the progression of acute joint inflammation into chronic destructive arthritis has also been demonstrated in an IL-17 receptor deficient (IL-17R-/-) mouse model [25]. These IL-17R-/- mice showed suppressed joint inflammation and impaired synovial expression of IL-1 and MMPs. A number of studies have linked IL-17A producing Th17 cells to bone destruction [13,26].

IL-17A appears to be associated with chronicity as demonstrated by murine models of collagen-induced arthritis. IL-17A was strongly dependent on TNF-α in the early stages of experimental arthritis; however, at a later stage the disease became IL-17A driven and both TNF-α and IL-1 independent [27]. Th17 cells were implicated in the erosive stage of chronic arthritis independent of TNF-α [28]. In human RA peripheral blood mononuclear cells the IL-17A/TNF-α ratio at baseline was found to be lower in responders to anti-TNF therapy as opposed to nonresponders [29]. This observation provides further evidence for IL-17A having a role in disease chronicity in human RA.

The aim of this study was to further examine the expression of IL-17A in actively inflamed joints and to elucidate the mechanism of IL-17A, alone and in combination with TNF-α and oncostatin M (OSM) in matrix turnover and cartilage degradation using whole RA synovial tissue explants, primary synovial fibroblasts (RASFC) and normal human cartilage cultures. Furthermore, we examined the relation of IL-17A to matrix turnover in patients following anti-TNF-α therapy.

## Materials and methods

### Synovial tissue, serum and synovial fluids

All patients fulfilled the 1987 American College of Rheumatology criteria for a diagnosis of RA [30] and had actively inflamed knee joints. RA ST was obtained at arthroscopy, using local anaesthesia as previously described [31]. SF was obtained from 49 patients with inflammatory arthritis (RA, n = 29; Spondyloarthropathy (SpA), n = 20) and five OA patients by arthrocentesis and stored at -80°C. Paired serum was obtained in 45 of 49 of these patients and serum from eight healthy volunteer controls was obtained. Fully informed written consent was obtained from each patient and the study was approved by the St. Vincent's University Hospital Ethics and Medical Research Committee.

### Preparation of synovial tissue lysates

ST obtained at arthroscopy (RA, n = 11, psoriatic arthritis (PsA), n = 12, OA, n = 3) were snap frozen in liquid nitrogen and homogenised using a Mikro-Dismembrator (B. Braun Biotech International, Allentown, Pennsylvania, USA). Homogenised samples were resuspended in protein lysis buffer and stored at -80°C. Protein concentration was determined by the BCA protein assay (Pierce, Rockford, IL, USA).

### Quantification of IL-17A protein levels

IL-17A protein expression was measured in serum, SF and ST lysates by specific ELISA (R&D Systems Europe, Abingdon, Oxon, United Kingdom). The ELISA standard curve range was 15 pg/ml to 1000 pg/ml. The lowest standard was used as the detection limit. IL-17A expression in synovial tissue lysates was corrected for total protein concentration.

### IL-17A Immunohistochemistry

Biopsy samples obtained at arthroscopy were embedded in Tissue Tek medium and then snap-frozen and stored in liquid nitrogen until sectioned for analysis. Serial 7 μM microtome sections were mounted on Superfrost slides and fixed in acetone for 10 minutes. The sections were then washed and blocked with 1 × casein for 30 minutes. The sections were then washed and incubated with 2 μg/ml goat polyclonal anti-IL-17A (Santa Cruz, Heidelberg, Germany) or normal immunoglobulin (Ig) G2a control. After incubation for one hour at room temperature and washing, the sections were incubated with biotinylated mouse anti-goat IgG antibody for 30 minutes at room temperature, followed by strepavidin-peroxidase complex (Dako, Glostrup, Denmark) for 30 minutes and 3,3'-diaminobenzidine tetrahydrochloride for five minutes. Nuclear counterstaining was performed using Mayer's haematoxylin, the sections were then dehydrated, and mounted in Dibutyl Phthlate Xylene (DPX). Sections from 11 RA and 3 OA patients and healthy control tissue (n = 1) were analysed for IL-17A synovium expression.

### Primary fibroblast cell culture

RASFC were obtained by enzymatic digestion of synovial biopsy specimens with 1 mg/ml of type 1 collagenase (Worthington Biochemical, Lakewood, New Jersey, USA) in RPMI (Gibco BRL, Warrington, UK) for four hours at 37°C in humidified air with 5% carbon dioxide. Dissociated cells were plated in RPMI 1640 supplemented with 10% FCS (Gibco BRL, Warrington, UK), 10 ml of 1 mmol/L HEPES (Gibco BRL, Warrington, UK), penicillin (100 units/ml), streptomycin (100 units/ml), and fungizone (0.25 μg/ml) (all from Biosciences, Co. Dublin, Ireland). The cells were incubated and grown to confluence in T75 flasks (about 10 days) at 37°C in humidified air with 5% carbon dioxide before being harvested with trypsin and passaged. RASFs between the fourth and eighth passages were used for experiments.

### Primary chondrocyte cell culture

Normal human articular cartilage was obtained from patients undergoing surgery for traumatic fracture of the femoral neck, each of whom had no history or radiological evidence of arthritis. Chondrocytes were isolated from the tissue by sequential proteolysis [32]. Cells were plated in DMEM supplemented with 10% FCS, 10 ml of 1 mmol/L, penicillin (100 units/ml), streptomycin (100 units/ml) and fungizone (0.25 μg/ml). Chondrocytes were used for experiments up until the eighth passage.

### RASFC, cartilage and chondrocyte cultures

Cartilage explant cultures were prepared using 3 mm punch biopsy specimens, thus ensuring that only full-depth cartilage biopsy samples were used. RASFC and cartilage explants were cultured in 96-well plates in serum-free RPMI 1640 supplemented with 10% FCS, penicillin (100 units/ml), and streptomycin (100 units/ml) in the presence of TNF-α (10 ng/ml), OSM (10 ng/ml) and IL-17A (50 ng/ml) alone and in combination. Cartilage explants were cultured over a time course of 15 days, which has been demonstrated previously to be an optimal time-point to examine proteoglycan depletion from cartilage sections [33,34]. As RASFCs are the primary cells that invade cartilage, RASFC experiments were also performed over a 15-day time course to examine if induction of MMPs in response to IL-17A could be sustained over the same time period. Culture supernatants were harvested every four days, and wells were replenished with fresh medium containing cytokine conditions that were identical to the medium used on day 1. Following the culture period, supernatants were pooled from each time point and stored at -80°C for analysis of proMMP-1, proMMP-13, TIMP-1, MMP-2 and MMP-9. Cartilage and RASFC experiments were performed in duplicate. Cartilage explants were paraffin embedded for immunohistological analysis.

Primary chondrocytes were cultured in 96-well plates in serum-free DMEM supplemented with penicillin (100 units/ml), and streptomycin (100 units/ml) for 24 hours in the presence of TNF-α (10 ng/ml), OSM (10 ng/ml) and IL-17A (50 ng/ml) alone and in combination. Chondrocyte experiments were performed in duplicate.

### Histological examination of human cartilage explants

Following the culture period, human cartilage explants were removed and fixed overnight in 7% formaldehyde in PBS (pH 7.4) and embedded in wax. Five-micrometer sections were stained with H&E and examined microscopically. For analysis of proteoglycans, 5 μm sections were stained with Safranin O-fast green and counterstained with haematoxylin [35].

### Whole RA synovial tissue explants

An *ex vivo *RA ST explant model was established, as previously described [36]. Each ST biopsy section was placed in a 96-well plate in serum-free RPMI supplemented penicillin (100 units/ml), streptomycin (100 units/ml), and fungizone (0.25 μg/ml) for 24 hours at 37°C in air with 5% carbon dioxide. Synovial explants were then stimulated (in triplicate) for 24 hours with TNF-α (10 ng/ml; R&D Systems, Europe, Abingdon, Oxon, United Kingdom) and IL-17A (10 to 20 ng/ml; R&D Systems, Europe, Abingdon, Oxon, United Kingdom). Following incubation for 24 hours, biopsy wet weights are obtained. The conditioned media was aspirated, collected and frozen at -80°C until assayed for proMMP-1, MMP-2, MMP-9 and TIMP-1 by ELISA and zymography. For Humira blockade experiments, each ST biopsy section was placed in a 96-well plate in full DMEM for 48 hours with Humira (10 μg/ml) or IgG control antibody (4 μg/ml). Following the incubation period, biopsy wet weights were obtained. The conditioned media was aspirated, collected and frozen at -80°. Supernatants were assayed for IL-17A by an MSD assay as this has a sensitivity of 0.4 pg/ml.

### ProMMP-1, proMMP-13, pro-MMP-3, TIMP-1 and TIMP4 quantification

ProMMP-1, proMMP-13 and TIMP-1 levels were quantified by specific ELISA (R&D Systems Europe, Abingdon, Oxon, United Kingdom). The ELISA minimum detectable doses were 0.021 ng/ml, 7.7 pg/ml, 0.009 ng/ml, 0.08 ng/ml and 4.91 pg/ml. The ELISA standard ranges were 10 ng/ml to 0.156 ng/ml, 5000 pg/ml to 78 pg/ml, 0.002 ng/ml to 0.045 ng/ml, 10 ng/ml to 0.156 ng/ml and 2.14 pg/ml to 10.0 pg/ml, respectively.

### Gelatin zymography for MMP-2 and MMP-9

Culture supernatants were separated by electrophoresis under nonreducing conditions by SDS-PAGE in 10% polyacrylamide gels copolymerised with 1% gelatin. Gels were washed vigorously twice for 25 minutes in 2.5% Triton X-100 to remove SDS, rinsed for 25 minutes in dH_2_O, then incubated overnight in 50 mM Tris/NaCl, pH 7.5, 10 mM CaCl_2 _at 37°C. Following overnight incubation gels were rinsed for five minutes in dH_2_O before addition of zymography stain (150 ml dH_2_O, 75 ml isopropanol, 25 ml acetic acid, 0.6255 g Brilliant Blue R). Gels were visualised using the UVP Bioimaging AutoChemi system (UVP, Cambridge, UK).

### Patients pre- and post-biologic therapy

A total of 38 patients were recruited from rheumatology outpatient clinics at St. Vincent's University Hospital and were followed up prospectively for one year. All patients had clinically active disease, with 28-joint count Disease Activity Scores (DAS28) of more than 3.2 points despite conventional disease-modifying anti-rheumatic drug therapy, and were offered treatment with biologic agents. Patients who had previously received biologic therapy were excluded from the study. Changes in conventional therapy were permitted during biologic therapy at the discretion of the patient's treating rheumatologist; however, no changes in the disease-modifying anti-rheumatic drug dosage were made during the study. Following approval by the institutional ethics committee at St. Vincent's University Hospital, all patients gave their fully informed written consent prior to inclusion in the study. All 38 patients began biologic therapy after their baseline assessment of disease activity. Patients were evaluated before and at 1, 3 and 12 months after initiation of biologic therapy.

Blood samples were obtained and sera was separated and stored at -80°C until used for biomarker analysis, and samples were available for this study at baseline and three months. Clinical evaluation at each assessment was performed using the DAS28, and the modified Health Assessment Questionnaire [9]. The DAS28 response was analysed both by changes in scores from baseline and by response categories according to the European League Against Rheumatism (EULAR) criteria [9]. A DAS28 response at three months was defined as a reduction in the DAS28 score of 0.6 points or more and a final DAS28 score of 5.1 points or less. A DAS28 nonresponse was defined as an improvement of less than 0.6 points or a final DAS28 score of more than 5.1 points. Patients achieving clinical remission at six months were identified according to EULAR criteria (DAS28 < 2.6 points) [9]. In addition, the patient's global assessment of his or her overall health was recorded at each visit, using a visual analog scale of 0 to 100 mm, where 0 is the best and 100 is the worst score. Serum was assessed for IL-17A, MMP-1, TIMP-1, MMP3, TIMP-4, acute serum-amyloid A (A-SAA), the collagen degradation markers C1, 2C and C2C and the synthesis markers CS846 and CPII at baseline and three months post therapy.

### Measurement of acute serum-amyloid A

A-SAA protein levels were measured using a sandwich enzyme immunoassay (Biosource, London, UK). Standards ranged from 9.4 to 300 ng/ml. The minimal detectable dose of the assay was 5 ng/ml.

### Quantification of IL-17A by MSD assay

Expression of IL-17A in synovial explant was assayed by MSD assay. The assay standard range was 0.15 pg/ml to 10,000 pg/ml. The lowest limit of detection was 0.4 pg/ml.

### Quantification of cartilage neoepitopes – C1, 2C, C2C, CS846 and CPII

The collagen degradation markers C1, 2C and C2C and the synthesis markers CS846 and CPII were measured by competitive immunoassay as per manufacturer's instructions (Ibex, Montreal, Canada). The ELISA minimum detectable doses were 0.03 μg/ml, 10 ng/ml, 20 ng/ml and 50 ng/ml. The ELISA standard ranges were 0.03 μg/ml to 10 μg/ml, 10 ng/ml to 1 μg/ml, 20 ng/ml to 1000 ng/ml and 50 ng/ml to 2000 ng/ml, respectively

### Statistical analysis

Statistical analysis was performed using SPSS 11 for Windows (SPSS, Chicago, IL, USA). Wilcoxon Rank and Mann Whitney U statistical tests were used. *P *values less than 0.05 were considered significant.

## Results

### Expression of IL-17A in the inflammatory joint

**Figure 1:**
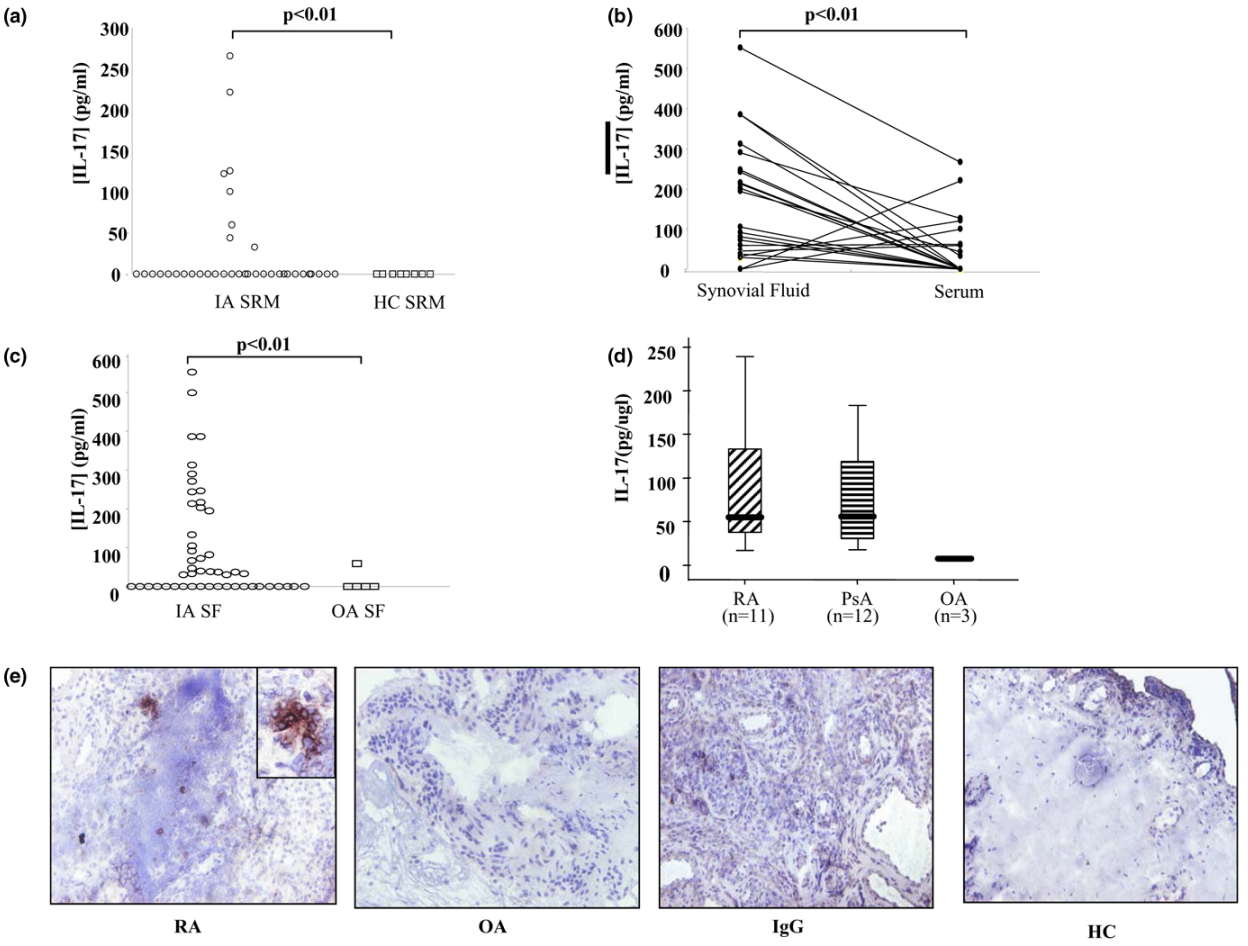
Over-expression of IL-17A in the human inflamed joint. IL-17A expression was measured by ELISA in serum (SRM) **(a) **from patients with inflammatory arthritis (IA; n = 40) vs. healthy controls (HC), **(b) **paired serums and synovial fluids (SF; n = 45), **(c) **synovial fluids from patients with inflammatory arthritis (n = 49) vs. osteoarthritis (OA) and **(d) **rheumatoid arthritis (RA; n = 11), psoriatic arthritis (PsA; n = 11) and OA (n = 3) synovial tissue lysates. **(e) **IL-17A-producing cells are detected in RA but not OA or healthy control synovium (E).

IL-17A levels were detectable in 54% of SF and 23% of serum. IL-17A serum levels were higher in patients with inflammatory arthritis compared with OA patients (24.3 ± 9.6 pg/ml vs. 12.32 ± 12.32 pg/ml). Serum IL-17A levels were significantly higher in patients with inflammatory arthritis compared with healthy controls (*P *< 0.05; Figure 1a). Furthermore, levels of IL-17A in SF were significantly higher than their matched serum levels (Figure 1b). SF IL-17A levels were also higher in inflammatory arthritis compared with OA (Figure 1c). High levels of IL-17A expression were demonstrated in ST lysates in both RA and PsA, markedly higher than OA ST (Figure 1d). No significant difference was found between levels in RA and PsA tissue. SF IL-17A levels were found to correlate directly with a measure of disease activity – C-reactive protein (CRP) (n = 43, r^2 ^= 0.330, *P *< 0.05) and disease duration (n = 24, r^2 ^= 0.470, *P *< 0.05). IL-17A was expressed in RA sublining, but not OA or healthy control synovium (Figure 1e). IL-17A expression tended to be scattered throughout the sublining; however, in some RA patients we demonstrated an aggregate of IL-17A positive cells (Figure 1e).

### IL-17A modulates MMP production

**Figure 2:**
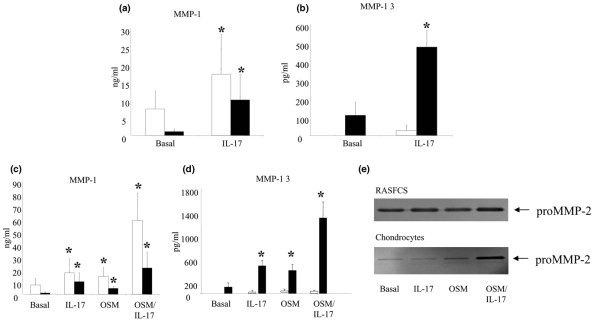
Stimulation of RASFCs and chondrocytes with IL-17A alone and combined with TNF-α and OSM causing significant matrix turnover. Primary synovial fibroblasts (RASFCs; white bars) and chondrocytes (black bars) were cultured in the presence of IL-17A (50 ng/ml) alone and in combination with TNF-α (10 ng/ml) or oncostatin M (OSM; 10 ng/ml) for 15 days and 24 hours respectively. **(a to d) **Culture supernatants were analysed for the expression of matrix metalloproteinase (MMP)-1 and MMP-13 by ELISA. Values are the mean and standard error results from nine experiments (RASFCs) and five experiments (chondrocytes). * *P *< 0.05 versus baseline. **(e) **MMP-2 activity was examined by gelatin zymography in the culture supernatants.

RASFC MMP-1 production was significantly increased by IL-17A (*P *< 0.015; Figure 2a) and for MMP-13 (*P *< 0.05; Figure 2b). Primary chondrocyte MMP-1 and MMP-13 expression was also significantly up-regulated by IL-17A (*P *< 0.05; Figure 2b). IL-17A and OSM combined potentiated the effect on MMP-1 and MMP-13 induction in both primary chondrocytes and RASFC compared with baseline and either cytokine alone (*P *< 0.05; Figures 2b, d). Similar effects were also observed with TNF-α (data not shown). Although no major effect was observed for MMP-2 activity in RASFCs, it was strongly upregulated by the combination of IL-17A/OSM in primary chondrocytes compared with either cytokine alone. (Figure 2e).

### IL-17A potentiates effects of TNF-α and OSM on cartilage degradation

**Figure 3:**
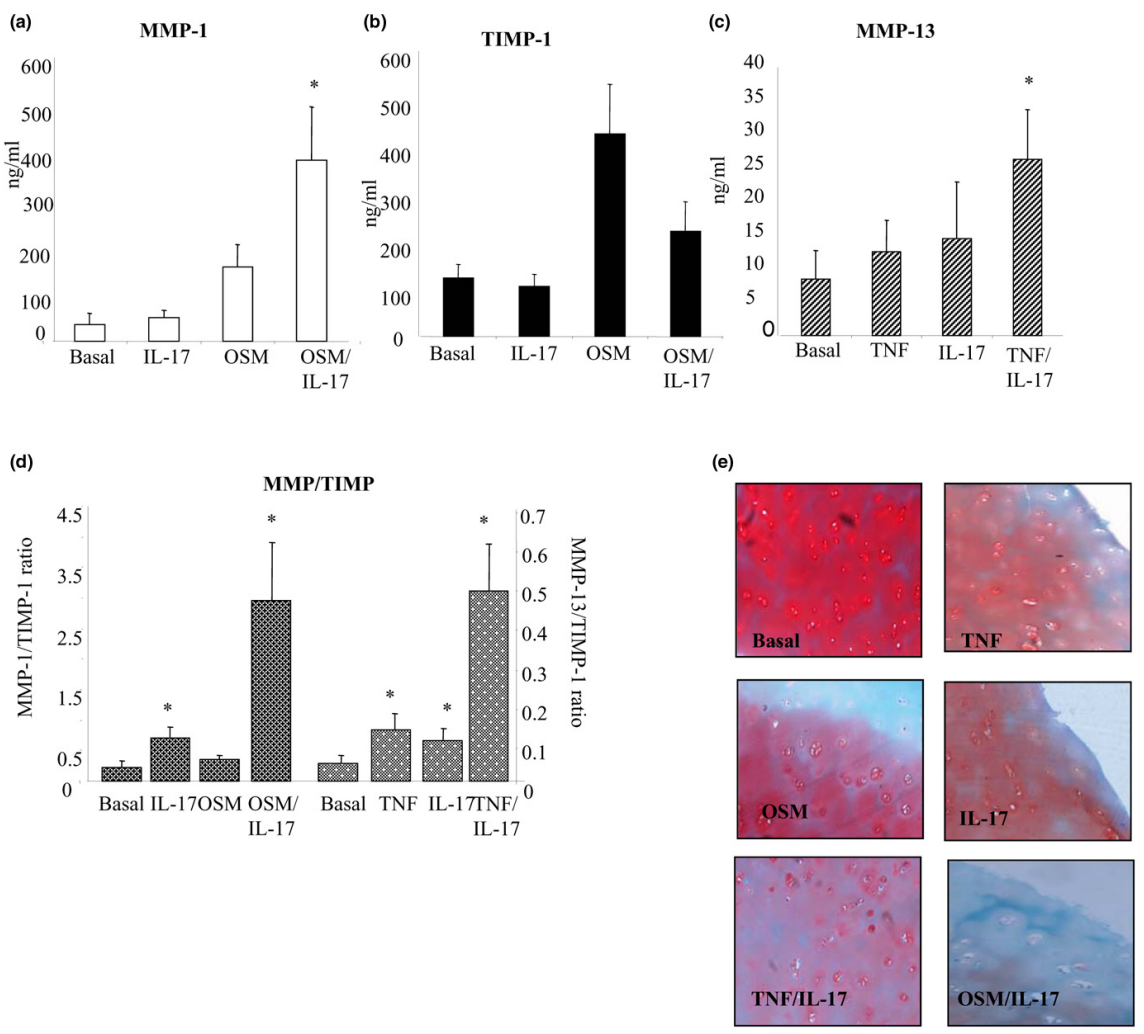
IL-17A in combination with TNF-α and OSM synergistically shifts the MMP-1: TIMP-1 and MMP-13: TIMP-1 ratios and drives cartilage destruction. Human cartilage explants were cultured in the presence of IL-17A (50 ng/ml) alone and in combination with TNF-α (10 ng/ml) or oncostatin M (OSM; 10 ng/ml). **(a to d) **Culture supernatants were analysed for the expression of matrix metalloproteinase (MMP)-1, MMP-13, and tissue inhibitor of metalloproteinase (TIMP)-1 by ELISA. Values are the mean and standard error results from nine experiments. * *P *< 0.05 versus baseline. Cartilage was formalin-fixed and embedded. **(e) **Proteoglycan staining was demonstrated by safranin O-fast green immunostaining.

IL-17A upregulates MMP-1 production in human cartilage cultures (Figure 3a); however, this did not reach significance. OSM significantly upregulated MMP-1 production in cartilage explants (*P *< 0.01). In combination IL-17A significantly potentiated the effect of OSM on MMP-1 production compared with basal (*P *< 0.01) and either cytokine alone (*P *< 0.05; Figure 3a). No significant effect was seen on TIMP-1 production (Figure 3b). The combination of IL-17A and OSM significantly shifts the MMP-1: TIMP-1 ratio in favor of cartilage degradation compared with baseline and either cytokine alone (*P *< 0.01; Figure 3d). A similar effect was seen on the MMP-13/TIMP-1 ratio in cartilage (*P *< 0.05; data not shown). Cartilage MMP-13 production was also upregulated by IL-17A and TNF-α alone, from basal of 8.34 ± 4.39 ng/ml to 14.47 ± 8.50 ng/ml and 12.56 ± 4.6 ng/ml, respectively (Figure 3c); however, this was not significant. The combination of IL-17A and TNFα had a potentiation effect significantly up-regulating MMP-13 production (*P *< 0.01; Figure 3c). The MMP-13: TIMP-1 ratio was also significantly shifted by the combination of TNF-α and IL-17A compared with basal (*P *< 0.01) and either cytokine alone (*P *< 0.05) (Figure 3d). Cartilage incubated with combinations of TNF-α/IL-17A and OSM/IL-17A, demonstrated almost complete proteoglycan depletion as shown by Safranin-O staining whereas the cytokines alone showed only a mild reduction (Figure 3e).

### IL-17A stimulation alters MMP-1: TIMP-1 ratio in whole RA ST explants

**Figure 4:**
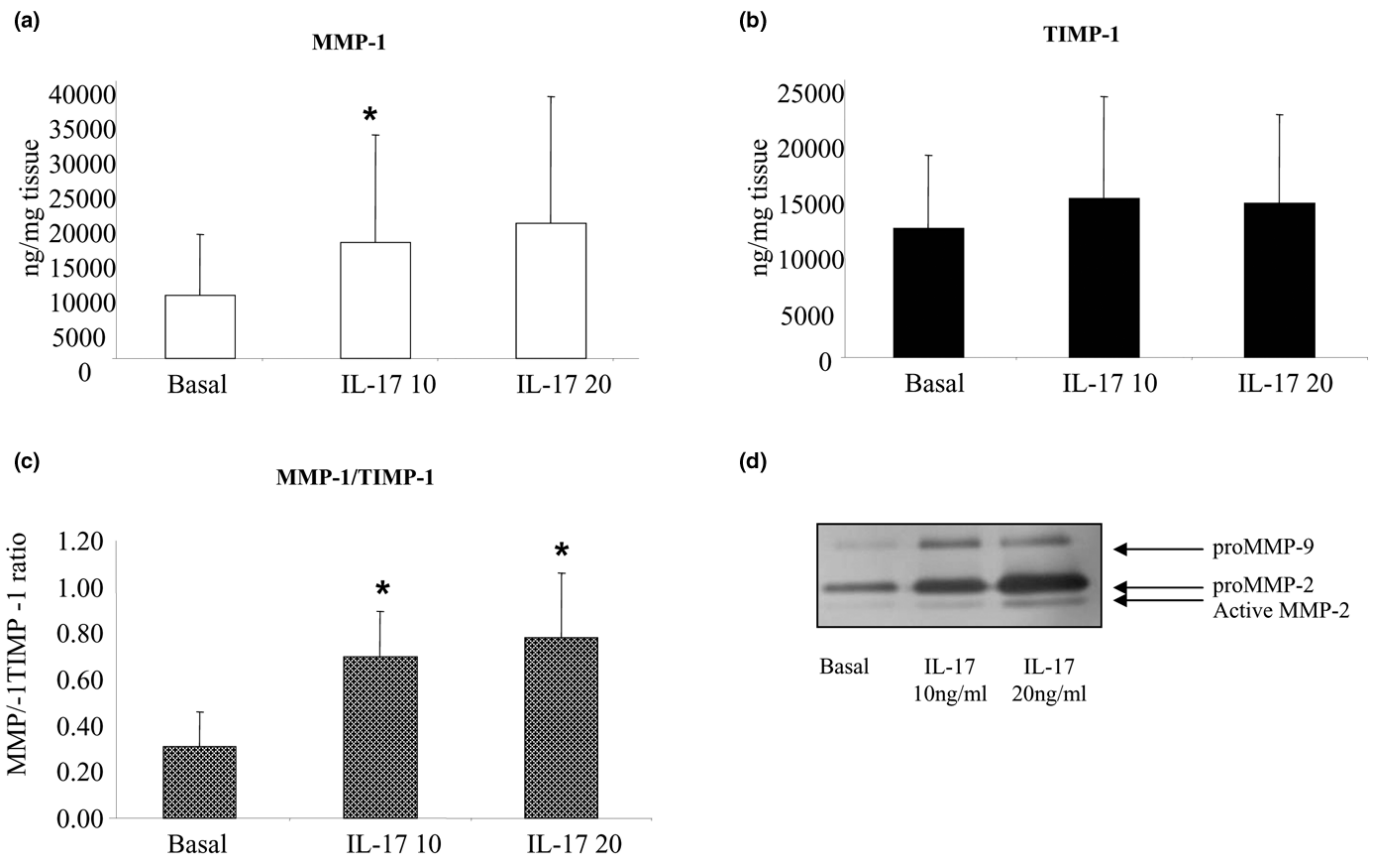
IL-17A significantly shifts the MMP-1: TIMP-1 ratio in rheumatoid arthritis synovial explant cultures. Whole rheumatoid arthritis (RA) synovial tissue explants were serum-starved for 24 hours and incubated with TNF-α (10 ng/ml), IL-17A (10 ng/ml) or IL-17A (20 ng/ml) alone and in combination. **(a to c) **Levels of matrix metalloproteinase (MMP)-1 and tissue inhibitor of metalloproteinase (TIMP)-1 in culture media were measured by ELISA. Values are the mean and standard error results from seven experiments. * *P *< 0.05 versus baseline (Basal). **(d) **MMP-2 and -9 activity in the culture supernatants was assessed by gelatin zymography.

RA ST explants (n = 11) were cultured in the presence of IL-17A (10 ng/ml or 20 ng/ml) or TNF-α (10 ng/ml), nine showed a response to stimulation with both IL-17A and TNF-α. MMP-1 production by ST explants was increased 1.8 and 2.1 fold by IL-17A (10 and 20 ng/ml) and 4.4 fold by TNF-α from basal of 9202.64 ± 8806.31 ng/mg of tissue to 16,711.80 ± 15,535.07 ng/mg of tissue (*P *< 0.05), 19,510.08 ± 18,261.87 ng/mg of tissue (*P *= 0.066) and 40,884.073 ± 3,5621.206 ng/mg of tissue (*P *< 0.01), respectively (Figure 4a). No significant effect was observed for TIMP-1 production following stimulation with both concentrations of IL-17A or TNF-α (Figure 4b). However, a significant shift in the MMP-1: TIMP-1 ratio was demonstrated (*P *< 0.01). Furthermore increased expression of pro-MMP-9 and both the pro and active forms of MMP-2 were demonstrated in response to IL-17A stimulation (Figure 4d).

### Regulation of IL-17A expression by biologic therapy

IL-17A protein levels were measured in a previously described cohort of patients [9] undergoing biologic therapy by ELISA. Baseline and three month serum samples of 38 patients were assayed. At baseline, nine patients showed detectable levels of IL-17A. Following three months of therapy, two patients whom at baseline showed undetectable levels had detectable levels of IL-17A. These 11 patients were categorised as the IL-17A-positive group. The remaining 27 patients were negative for IL-17A expression at both baseline and three months and were categorised as the IL-17A-negative group, 70% of which had a clinical response to anti-TNF-α therapy at three months.

**Figure 5:**
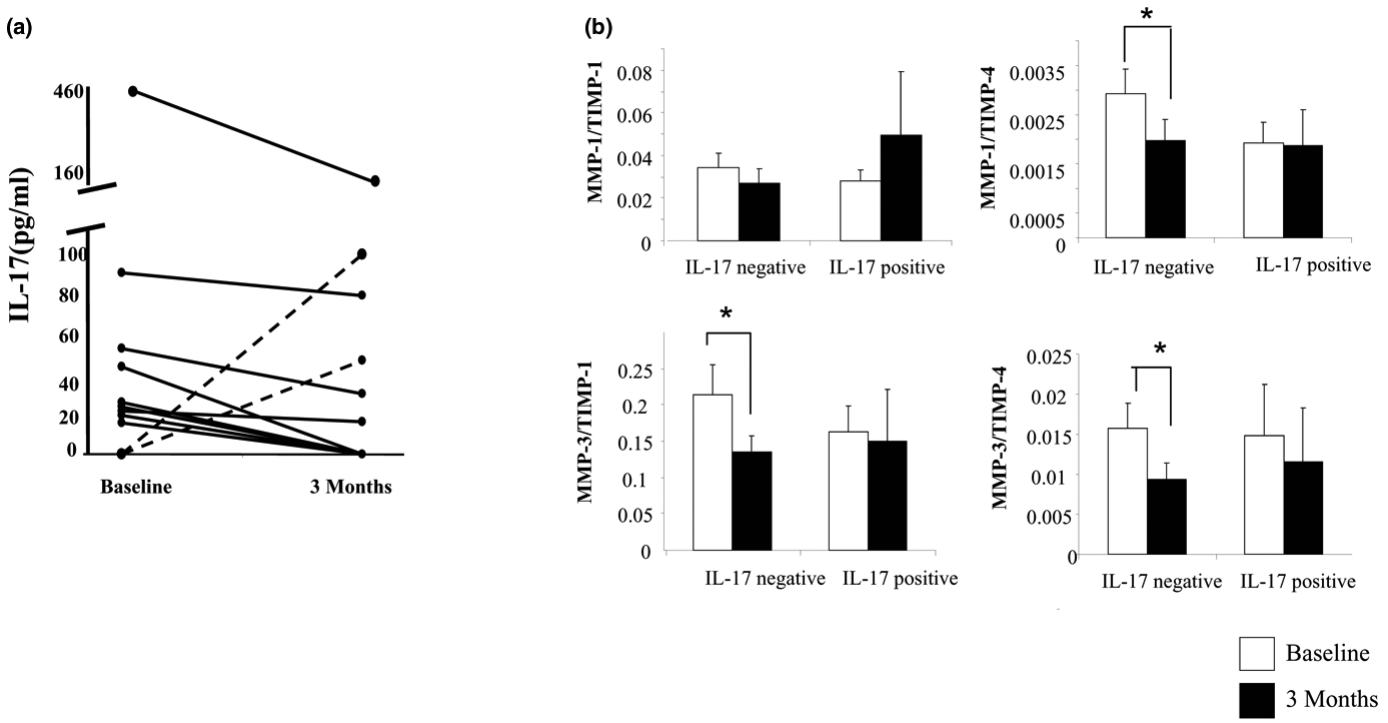
IL-17A expression is modulated pre/post biologic therapy. Baseline and three months serum samples from 38 patients were analysed using IL-17A ELISA. Eleven of these patients showed detectable levels of IL-17A. **(a) **Data shown are of these 11 patients. **(b) **Matrix metalloproteinase (MMP)/tissue inhibitor of metalloproteinase (TIMP) ratios are significantly reduced in IL-17A-negative patients three months following biologic therapy.

In the IL-17A-positive group, 82% showed a decrease in IL-17A levels after three months of therapy, while 18% showed an increase. Patients that showed a decrease post therapy were also those patients that had been defined as clinical responders, while those showing an increase post therapy (dotted lines) were non-responders (Figure 5a). All but one of these patients maintained this response to 12 months post therapy. The change in IL-17A levels pre/post biologic therapy strongly correlated with the change in CRP (r^2 ^= 0.817, *P *< 0.01) and the change in A-SAA (r^2 ^= 0.627, *P *< 0.05), markers of the acute phase response. When patients were categorised into those with detectable IL-17A levels and those with no detectable IL-17A levels, a significant difference in serum matrix turnover markers was demonstrated. MMP-1 and MMP-3 levels were significantly reduced in the IL-17A-negative patients (*P *< 0.05) compared with the IL-17A-positive patients, which showed no significant change (data not shown). The MMP-1/TIMP-1 ratios were higher in the IL-17A-negative patients compared with IL-17A-positive patients but this was not significant (Figure 5b). A significant reduction was demonstrated for the MMP-1/TIMP4, MMP3/TIMP1 and MMP3/TIMP4 ratios in IL-17A-negative patients (all *P *< 0.05), compared with IL-17A-positive patients where we demonstrated no significant reduction in any of the MMP/TIMP ratio. This demonstrates decreased matrix degradation three months post therapy in those patients with no detectable IL-17A levels (Figure 5b). Although there is no change from baseline MMP/TIMP ratios pre/post anti-TNF-α therapy in the IL-17A-positive patients, the levels of IL-17A were decreased post therapy. This suggests that patients with detectable IL-17A levels may sustain a higher MMP/TIMP ratio than those that are negative; however, other complex regulatory processes may also be involved in regulating matrix turnover, possibly through interactions with IL-17A or independently. Cartilage biomarkers were also measured in the IL-17A-negative and positive patients. There were no significant differences in the serum levels of C1, 2C, C2C and CPII pre/post therapy in either group. However, CS846 a marker of proteoglycan synthesis was significantly higher in the IL-17A-positive patients compared with the IL-17A-negative group at both baseline (262.5 ± 68.1 ng/ml vs. 158 ± 18.4 ng/ml) and three months post therapy (246.2 ± 60 ng/ml vs. 159 ± 14.9 ng/ml; *P *< 0.05). Finally, IL-17A production was measured in synovial explants following incubation with Humira and IgG control antibody. Levels of IL-17A were too low to be detected, or were at the lower end of the standard curve and were not reliable.

## Discussion

In this study we examined the expression of IL-17A in the human inflammatory joint. An *ex vivo *RA ST explant model, RASFCs, normal human cartilage and chondrocyte cultures were used to investigate the mechanistic role of IL-17A, alone and in combination, with TNF-α and OSM on matrix turnover and cartilage proteoglycan release. The effect of biologics therapy on IL-17A expression and matrix turnover was also examined *in vivo *in a previously described cohort.

In this study we have shown high expression of IL-17A in ST lysates with no significant difference between RA and PsA tissue. Furthermore, IL-17A expression was markedly higher in RA and PsA ST compared with OA. Our results are consistent with previous studies demonstrating increased IL-17A production from RA patients compared with OA [13]; however, our data differ as samples are from patients with early active inflammation before commencing biologic therapy. We also report significantly higher IL-17A levels in SF and serum from inflammatory arthritis patients compared with OA and healthy controls. SF levels of IL-17A were significantly higher than matched serum suggesting that the cytokine is predominantly produced locally in the inflamed joint. Although previous reports showed elevated serum and SF IL-17A levels in RA patients compared with healthy controls [37] these studies did not examine matched serum/SF samples as performed in this study. Interestingly, there was a strong correlation of SF IL-17A levels with CRP and disease duration. This finding is not surprising because previous animal model studies have suggested IL-17A drives disease activity and is associated with disease chronicity [27]. Furthermore, IL-17A is a potent inducer of CRP from human smooth muscle cells and hepatocytes [38]. We also demonstrated IL-17A expression in RA but not OA or healthy control synovium. IL-17A was expressed in 70% of RA patients examined, which is consistent with previous reports [39].

A fine balance exists between active MMP and TIMP levels in normal tissue and cartilage. In inflammatory conditions such as RA, this balance shifts leading to an increase in the ratio of active MMP: TIMP [40]. The effect of IL-17A on MMP expression was first examined in RASF and normal human chondrocyte cultures. IL-17A alone upregulated the expression of MMP-1 and MMP-13 in both chondrocytes and RASFCs. IL-17A combined with OSM synergistically upregulated MMP-1 production in both chondrocytes and RASFs. A similar effect was observed for chondrocyte MMP-13 production with a less potent effect on RASF MMP-13 production. IL-17A alone also caused an increase in matrix turnover in chondrocytes as seen by the increased MMP-9 activity. IL-17A combined with OSM had an additive effect on matrix turnover as seen by the increased MMP-9 activity compared with either cytokine alone. Our results are consistent with previous studies showing IL-17A induced MMP expression in human OA chondrocytes [41] and in RA synoviocytes [18]. In animal cells IL-17A has been previously shown to act synergistically with TNF-α and OSM in upregulating proinflammatory cytokine and chemokine expression and cartilage breakdown [18,42,43]. In this study for the first time we have shown in human cells isolated from patients with inflammatory arthritis that IL-17A combined with OSM synergistically upregulates the expression of MMP-1, MMP-2, MMP-9 and MMP-13 in both chondrocytes and RASFs.

We also showed for the first time in human cartilage explants and a human *ex vivo *synovial explant culture model, that IL-17A regulates MMP production and cartilage degradation, which is supported by previous animal studies [18,19]. IL-17A alone significantly shifted the MMP-1: TIMP-1 ratio in favour of a destructive pattern. Significant matrix turnover was also demonstrated by increased MMP-2 and MMP-9 activity in response to IL-17A stimulation. Mild proteoglycan depletion was observed in response to IL-17A stimulation visualised by loss of safranin-O staining. However, when IL-17A was combined with other key pro-inflammatory cytokines it significantly potentiated the effect of TNF-α and OSM on MMP-1 and MMP-13 production. This had a profound effect on the MMP/TIMP ratios, which shifted dramatically in favour of matrix degradation. Furthermore, when human cartilage explants were incubated with IL-17A, TNF or OSM alone, mild proteoglycan depletion was observed; however, when IL-17A was combined with OSM or TNF, near complete proteoglycan depletion compared with either cytokine alone was demonstrated.

These results suggest that in the inflamed joint environment, which has a complex milieu of pro-inflammatory cytokines, that in addition to exerting its effects alone, IL-17A appears to have an important role in dramatically potentiating the destructive effects of other pro-inflammatory cytokines, such as TNF-α and OSM. Increased MMP expression has been observed in human chondrocytes in response to IL-17A stimulation [41] but no previous studies has examined its effect on RA whole tissue synovial explants or human cartilage explants. Whole tissue explant cultures more closely mimic the joint environment, as the architecture, cell-cell interactions and ECM remains intact, which can result in expression of genes and proteins to stimuli that differ from monolayer cultures. Thus the combination of mono-culture and whole tissue explant culture in this study dissects the cellular responses such as MMP expression to specific stimuli more effectively [44].

Finally, we examined IL-17A and MMP/TIMP production in patients pre/post biologic therapy on IL-17A. We demonstrate for the first time that IL-17A serum levels are reduced in inflammatory arthritis patients following TNF blockade *in vivo*. We show that serum IL-17A levels are modulated by biologic therapy with 80% of patients showing a decrease in IL-17A three months post therapy. Significantly, this reduction was demonstrated in patients who showed a clinical response, while non-responders showed an increase. Change in IL-17A also correlated with change in CRP and A-SAA, markers of systemic inflammation. Previous studies have shown synovial membrane mRNA levels of IL-17A may predict joint damage progression in RA [45] and IL-17A serum levels correlate with disease severity in psoriasis patients [46]. IL-23 (a key factor in Th17 differentiation) has been genetically linked to increased susceptibility to psoriasis [46,47]. Furthermore, disease resolution in psoriasis patients following TNF blockade correlates with reduced Th17 responses [48].

Furthermore, we demonstrate a significant difference in MMP/TIMP ratios from baseline to three months post therapy in IL-17A-negative patients, with no significant difference in IL-17A-positive patients. However, interpretation of this data is complex. IL-17A-positive patients may exhibit a sustained stimulation of MMPs; however, as the IL-17A levels do reduce after three months in most patients, it may suggest other pro-inflammatory mediators such as TNF-α, IL-1β and OSM are driving MMP expression. Indeed, this is supported by our *in vitro *data and the results of other studies [34,42] demonstrating IL-17A acts in synergy with TNF-α, OSM and other cytokines leading to increased MMP activity.

Numerous studies have associated MMP serum levels in particular MMP-3 levels to disease activity and radiographic progression [49-55]. SNPs in the IL-17A gene have been associated with radiographic progression [56]. Furthermore, one of the main drivers of IL-17A production, IL-23, is present in higher levels in patients with bone erosions than those without erosions [57]. The observation of higher CS846 levels in IL-17A-positive sera may be relevant radiographically. Increased serum levels of CS846 are an indicator of increased turnover of newly formed matrix, as part of an attempt to repair cartilage degradation [58]. Furthermore, patients with rapid radiographic progression have been shown to have higher CS846 epitope levels than slow progressors [59].

Overexpression of IL-17A by injection or gene expression significantly increased inflammatory infiltrate and resulted in extensive joint destruction [22,60]. Furthermore, in studies that blocked IL-17A or in IL-17R-/- mice reduced inflammation and bone damage was observed [61,62]. In murine studies IL-17A has been shown to act independently of TNF-α [27,28]. A number of studies have shown TNF inhibition has no effect on IL-17A or IL-23 expression [63-65]. Furthermore, the IL-17A/TNF-α ratio pre-treatment was shown to be lower in responders to anti-TNF treatment [29]. This data and previous studies suggest that IL-17A synergises with other pro-inflammatory cytokines but can also enhance inflammation and destruction independently and would propose IL-17A as a potential target in the treatment of RA.

## Conclusions

In this study we have shown that IL-17A is highly expressed in the inflammatory joint and drives disease activity, implicating it as a key cytokine and potential therapeutic target. We have shown that IL-17A not only drives the proinflammatory response but also enhances the effect of TNFα and OSM, promoting increased destruction in the RA joint. Finally, we demonstrate that IL-17A levels are modulated *in vivo*, following anti-TNF therapy, and may reflect changes in matrix turnover.

## Abbreviations

A-SAA: acute serum-amyloid A; CRP: C-reactive protein; DAS28: 28-joint count Disease Activity Score; DMEM: Dulbecco's modified Eagle's medium; DPX: Dibutyl Phthlate Xylene; ECM: extracellular matrix; ELISA: enzyme-linked immunosorbent assay; EULAR: European League Against Rheumatism; FCS: fetal calf serum; H&E: haematoxylin and eosin; IG: immunoglobulin; IL: interleukin; MMP: matrix metalloproteinase; OA: osteoarthritis; OSM: oncostatin M; PBS: phosphate-buffered saline; PsA: psoriatic arthritis; RA: rheumatoid arthritis; RASF: RA synovial fibroblasts; RASFC: primary synovial fibroblasts; SF: synovial fluid; SpA: Spondyloarthropathy; ST: synovial tissue; TGF: transforming growth factor; TIMP-1: tissue inhibitor of metalloproteinase 1; TNF: tumour necrosis factor.

## Competing interests

The authors declare that they have no competing interests.

## Authors' contributions

EM performed most of the experiments, data analysis and manuscript preparation. RM collected the clinical cohort and participated in study design and data analysis. JMcC, MC and OS performed some experiments. BB and OF participated in study design and data analysis. UF and DV conceived the study and developed the study design, performed data analysis and prepared the manuscript. All authors read and approved the final manuscript.
